# Dengue Virus in Bats from Córdoba and Sucre, Colombia

**DOI:** 10.1089/vbz.2018.2324

**Published:** 2019-09-26

**Authors:** Alfonso Calderón, Camilo Guzmán, Salim Mattar, Virginia Rodriguez, Caty Martínez, Lina Violet, Jairo Martínez, Luiz Tadeu Moraes Figueiredo

**Affiliations:** ^1^ Department of Bacterilogy, Faculty of Health Sciences, University of Córdoba, Montería, Colombia.; ^2^ Doctorate in Tropical Medicine SUE-Caribe, University of Córdoba, Monteria, Colombia.; ^3^ Institute of Virology, Faculty of Medicine, University São Paulo, Ribeirão Preto, Brazil.; ^4^ Institute of Virology, University Sao Paulo, Ribeirao Preto, Brazil.

**Keywords:** *Chiroptera*, flavivirus, host, infection, zoonoses

## Abstract

Natural infection of dengue virus (DENV) in bats is an unexplored field in Colombia. To detect the presence of DENV in bats, a descriptive prospective study using a nonprobabilistic sampling was carried out; 286 bats in 12 sites were caught. Sample tissues of different animals were obtained; the RNA was obtained from tissues and a nested-RT-PCR was carried out and detected amplicons of 143 fragment of the NS5 gene were sequenced by the Sanger method. In nonhematophagous bats *Carollia perspicillata* and *Phyllostomus discolor* captured in Ayapel and San Carlos (Córdoba), respectively, an amplicon corresponding to NS5 was detected. The amplicons showed a high similarity with serotype-2 dengue virus (DENV-2). This is the first evidence of the DENV-2 genome in bats in from the Colombian Caribbean.

## Introduction

Emerging and re-emerging infectious diseases have now become among the most serious threats to public health. Approximately 75% of the diseases that have emerged during the past two decades have wild reservoirs (Woolhouse and Gowtage 2005, Jones et al. 2008). In this sense, bats are hosts of high viral diversity with high zoonotic potential worldwide (O'Shea et al. 2014, Calderon et al. 2016).

Some flavivirus such as dengue virus (DENV), Koyose virus (YOKV), Tamana virus (TABV), Rio Bravo virus (RBV), Japanese encephalitis virus (JEV), and West Nile virus (WNV) have been detected in bats (Bunde et al. 2006, Jiang et al. 2015, Tajima et al. 2015, Thompson et al. 2015, Abudes-Gallegos et al. 2018). Many flaviviruses can cause diseases in humans such as from a nonspecific febrile syndrome, triggering encephalitis, to hemorrhagic fever and death (Feitoza et al. 2017, Hall et al. 2017, Mattar et al. 2017). Dengue fever is a disease of great prevalence in tropical countries. Although bats can harbor many infectious viruses, they do not develop apparent signs of disease from any virus (Calderon et al. 2016). It is believed that the increase in the body temperature of bats as a result of flight increases the metabolic rate, the mitochondrial activity that triggers the immunological cascade, and the production of interleukins and prostaglandins, which would prevent them from being infected (Krysko et al. 2011, Wang et al. 2011, O'Shea et al. 2014, Mattar and Gonzalez 2016). The objective of this study was to establish a natural infection of DENV in bats from departments of Córdoba and Sucre (Colombia).

## Materials and Methods

### Study type and sampling

**FIG. 1.**
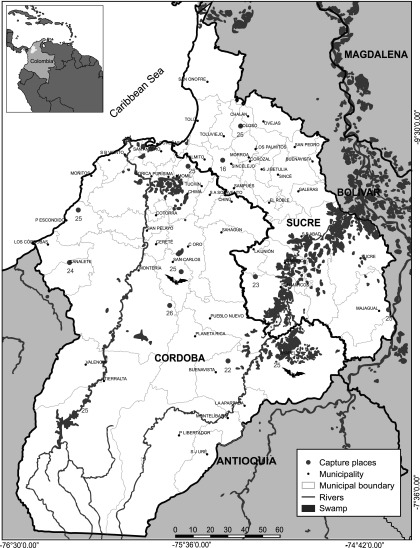
Capture sites of bats in Córdoba and Sucre (Colombia).

A descriptive prospective study using a nonprobabilistic sampling was carried out; 286 bats in 12 sites were caught: 8 in Córdoba and 4 in Sucre, both departments included the main ecosystems; these departments are located in the Caribbean area of Colombia (Fig. 1). The study was approved by the ethics committee of the Faculty of Veterinary Medicine of the University of Cordoba, Colombia; the committee took into account the instructions for researching with non-commercial purposes animals of the National Environmental Authority of Colombia. The bats were captured with the use of mist nets; they were later identified using dichotomous taxonomic keys (Linares 1998). Specimens listed as endangered species and pregnant or lactating females were released. Euthanasia was performed using an overdose of sodium pentobarbital. Brain, heart, lung, liver, kidney, and spleen were collected. The organs were stored in cryovials with Trizol™ (Invitrogen, Carlsbad, CA) and kept in liquid nitrogen.

### Molecular detection

RNA extraction was performed with Trizol (Invitrogen). The aliquots were suspended in 150 μL of nuclease-free water. The concentration of the RNA obtained from each organ was then measured using the NanoDrop 2000 (Spectrophotometers™). The cDNA synthesis was carried out with the reverse transcriptase enzyme M-MLV (Invitrogen) using random primers, as recommended by the manufacturers.

A nested RT-PCR using in the first round (Flavi 1+: 5′-GAYYTIGGITGYGGIGIGGIRGITGG-3′ and Flavi 1−: 5′-TCCCAICCIGCIRTRTCRTCIGC-3′) was performed; the nested with by Flavi 2+ (5′-YGYRTIYAYAWCAYSATGGG-3′) and Flavi 2− (5′-CCARTGITCYKYRTTIAIRAAICC-3′) was performed. As previously described, the set of primers amplifies products of 1360bp and, 143bp respectively (Sanchez et al. 2005). As a control of species, complementary primers were used to sequence a mitochondrial gene *mt* DNA from bats (Ramírez et al. 2014). As a positive control, the Yellow Fever Virus (YFV) vaccine prepared with an attenuated live virus of strain (17D-204-strain; Sanofi-Pasteur, Lyon-Francia), as a negative control water molecular biology grade was used. The amplicons obtained were sequenced by the Sanger method.

### Phylogenetic analysis

Using Clustal W, 2 sequences obtained in this study and 38 partial sequences of the NS5 gene were aligned with four DENV serotypes registered in GenBank. For the phylogenetic construction, the maximum likelihood method was used. The substitution model was Kimura of two parameters and to estimate the branch supports of each group, 10,000 pseudoreplications were produced; in total 100 positions were analyzed. The phylogenetic analysis was performed with MEGA X.

## Results

Table 1 shows the distribution of bats species by found sources.

**Table 1.** Distribution of Bats Species by Food Sources

*Food source*	*Captured species*	*No.*	*Food source*	*Captured species*	*No.*
Insectivorous	*Phyllostomus discolor*	42	Frugivorous	*Artibeus planirostris*	99
	*Molossus molossus*	14		*Carollia perspicillata*	38
	*Saccopteryx bilineata*	4		*Artibeus lituratus*	30
	*Eptesicus brasilensis*	1		*Sturnira lilium*	20
	*Rhogeessa yo*	2		*Carollia brevicauda*	1
	*Eumops glaucinus*	1		*Carollia castanea*	1
	*Lasiurus ega*	1		*Uroderma bilobatum*	11
	*Micronycteris microtis*	1	Piscivorous	*Noctilio albiventris*	3
	*Myotis nigricans*	1		*Noctilio leporinus*	3
	*Saccopteryx leptur*	1	Nectarivorous	*Glossophaga soricina*	6
	*Molossops temmincki*	1	Hematophagous	*Desmodus rotundus*	4
Omnivorous	*Trachops cirrhosus*	1			

Amplicons with a size of 143 bp of the NS5 gene of DENV in brain, lung, liver, and kidney of *Carollia perspicillata*, captured in Ayapel and *Phyllostomus discolor* in San Carlos (Córdoba) were detected. The sequences of these amplicons were deposited in the GenBank, the sequence obtained from *C. perspicillata* (CIIBT-106-2) with accession number MG011655.1, and the sequences of *P. discolor* (CIIBT-193-2) with the accession number MG011656.1.

The sequence MG011655.1 showed a similarity of 95.1% and coverage of 98.41% with the sequence FJ392595 of serotype-2 dengue virus (DENV-2) NS5 gene. The sequence MG011656.1 presented a similarity of 96.1% with coverage of 100% compared with the sequence FJ392595 of the GenBank. The results of the phylogenetic analysis show that the consensus sequence showed a high percentage of similarity with sequences of the gene coding for the NS5 protein of DENV-2. The phylogenetic analysis suggested the presence of DENV-2 in these tissues.

**FIG. 2.**
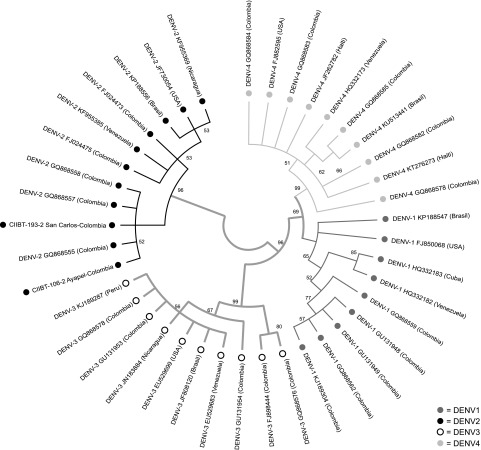
Phylogenetic tree by ML method; four clades are shown and the obtained sequences from Cordoba and Sucre bats are shown with additional *circles*. ML, maximum likelihood.

Four monophyletic clades were obtained, clearly differentiated that correspond to the four serotypes of DENV, each clade group individuals of the same serotype, and are separated by high values of branch support (Fig. 2). The sequences obtained, MG011655.1 and MG011656.1 (between two red circles), were integrated with sequences homologous to DENV-2 (shown with a single red circle).

## Discussion

Several investigations (Platt et al. 2000, Aguilar-Setien et al. 2008, Lavergne et al. 2009, Machain et al. 2013, Sotomayor-Bonilla et al. 2014, Vicente-Santos et al. 2017, Abudes-Gallegos et al. 2018) have demonstrated the presence of DENV in bats. In contrast, a study involving 240 wild bats from Campeche and Morelos (Mexico) indicated the absence of serological or molecular evidence of the DENV, the authors suggest that American bats could not be reservoirs or amplification host for DENV infection (Cabrera-Romo et al. 2016). That study included 19 *C. perspicillata* and there was no serological or molecular evidence of DENV; however, in this study we caught 38 *C. perspicillata*, and 1 from Ayapel showed partial sequences of the NS5 flavivirus gene.

The molecular evidence in this study agree with the conclusions of Marinkelle (1996), Platt et al. (2000), Aguilar-Setien et al. (2008), Lavergne et al. (2009), Machain et al. (2013), Hayman et al. (2013), Sotomayor-Bonilla et al. (2014), Schountz (2014), Moratelli and Calisher (2015), and Vicente-Santos et al. (2017) who clearly suggest that bats can be involved with different pathogens that affect human health.

In rural areas of Córdoba and Urabá (Colombia), mosquitoes *Aedes*, *Culex*, *Anopheles*, *Culiseta*, *Mansonia*, *Coquillettidia*, *Psorophora*, *Armigeres*, *Myzorhynchus*, and *Taeniothyncus* (Jaramillo et al. 2005, Parra-Henao and Suárez 2012) have been identified. A recent study in Córdoba has also reported DENV-2 in *Aedes aegypti*, YFV in *Haemagogus splenden*, SLEV in *Mansonia titillans* and *Culex* spp., and WNV in *Culex* spp. (Hoyos-López et al. 2016). These findings demonstrate that Córdoba is considered an endemic region for arboviruses and demonstrates an active circulation with risk to human health, denoting the importance of surveillance activities for emerging viruses (Mattar et al. 2005).

The transmission of DENV by mosquitoes could be limited in wild ecosystems, where urban species of *A. aegypti* or *Aedes albopictus* is found. It is believed that wild strains of DENV have low transmissibility or virulence for humans. Moreover, DENV has recently been found Diptera of the order Streblidae (*Strebla wiedemann* and *Trichobius parasiticus*) parasitizing bats, which is an interesting finding since flies could be involved in the maintenance of DENV in nature (Abundes-Gallegos et al. 2018).

The transmission of DENV to bats should involve the feeding of *Aedes* species (Kimpell 2013, Rey and Lounibo 2015). The presence of *A. aegypti* in all the municipalities of Córdoba (SDS 2015) and the high prevalence of the dengue cases in Córdoba (INS, 2016–2017) might be creating the ecoepidemiological conditions for the DENV to adapt to wild ecosystems. In Brazil, DENV infections were found in larvae of *A. albopictus* (transovarial transmission) and *Haemagogus leucocelaenus*, which could suggest a possible sylvatic cycle (De Figueiredo et al. 2010). Moreover, DENV has recently been found in Diptera order Streblidae (*S. wiedemann* and *T. parasiticus* parasiticus) parasitizing bats, which is an interesting finding since flies could be involved in the maintenance of DENV in nature (Abundes-Gallegos et al. 2018).

Further investigations are still needed to determine whether the DENV detected in *C. perspicillata* and *P. discolor* in this study is the result of an adaptation of the DENV to this particular ecological niche. Currently, the Asian genotype of DENV-2 is widely distributed in human populations and is expanding throughout the world. A possible explanation can be that bats, which are bitten by mosquitoes other than *Aedes*, act as reservoirs of DENV and could play a role in the epidemiology of DENV in tropical countries, where cities are close to forests.

## Conclusion

This study presents the first molecular evidence of the infection natural in bats captured in the Colombian Caribbean.
